# Contralateral cortico-ponto-cerebellar pathways reconstruction in humans *in vivo*: implications for reciprocal cerebro-cerebellar structural connectivity in motor and non-motor areas

**DOI:** 10.1038/s41598-017-13079-8

**Published:** 2017-10-09

**Authors:** Fulvia Palesi, Andrea De Rinaldis, Gloria Castellazzi, Fernando Calamante, Nils Muhlert, Declan Chard, J. Donald Tournier, Giovanni Magenes, Egidio D’Angelo, Claudia A. M. Gandini Wheeler-Kingshott

**Affiliations:** 1 Department of Physics, University of Pavia, Pavia, PV Italy; 2 Brain Connectivity Center, C. Mondino National Neurological Institute, Pavia, PV Italy; 3 Department of Electrical, Computer and Biomedical Engineering, University of Pavia, Pavia, PV Italy; 4 The Florey Institute of Neuroscience and Mental Health, Melbourne Brain Centre, Heidelberg, Victoria, Australia; 5 Florey Department of Neuroscience and Mental Health, University of Melbourne, Heidelberg, Victoria, Australia; 6 NMR Research Unit, Department of Neuroinflammation, Queen Square MS Centre, UCL Institute of Neurology, London, England United Kingdom; 7 School of Psychological Sciences, University of Manchester, Manchester, United Kingdom; 8 National Institute for Health Research, University College London Hospitals Biomedical Research Centre, London, United Kingdom; 9 Department of Biomedical Engineering, King’s College London, London, UK; 10 Centre for the Developing Brain, King’s College London, London, UK; 11 Department of Brain and Behavioral Sciences, University of Pavia, Pavia, Italy; 12 Brain MRI 3T Research Center, C. Mondino National Neurological Institute, Pavia, PV Italy

## Abstract

Cerebellar involvement in cognition, as well as in sensorimotor control, is increasingly recognized and is thought to depend on connections with the cerebral cortex. Anatomical investigations in animals and post-mortem humans have established that cerebro-cerebellar connections are contralateral to each other and include the cerebello-thalamo-cortical (CTC) and cortico-ponto-cerebellar (CPC) pathways. CTC and CPC characterization in humans *in vivo* is still challenging. Here advanced tractography was combined with quantitative indices to compare CPC to CTC pathways in healthy subjects. Differently to previous studies, our findings reveal that cerebellar cognitive areas are reached by the largest proportion of the reconstructed CPC, supporting the hypothesis that a CTC-CPC loop provides a substrate for cerebro-cerebellar communication during cognitive processing. Amongst the cerebral areas identified using *in vivo* tractography, in addition to the cerebral motor cortex, major portions of CPC streamlines leave the prefrontal and temporal cortices. These findings are useful since provide MRI-based indications of possible subtending connectivity and, if confirmed, they are going to be a milestone for instructing computational models of brain function. These results, together with further multi-modal investigations, are warranted to provide important cues on how the cerebro-cerebellar loops operate and on how pathologies involving cerebro-cerebellar connectivity are generated.

## Introduction

**Figure 1:**
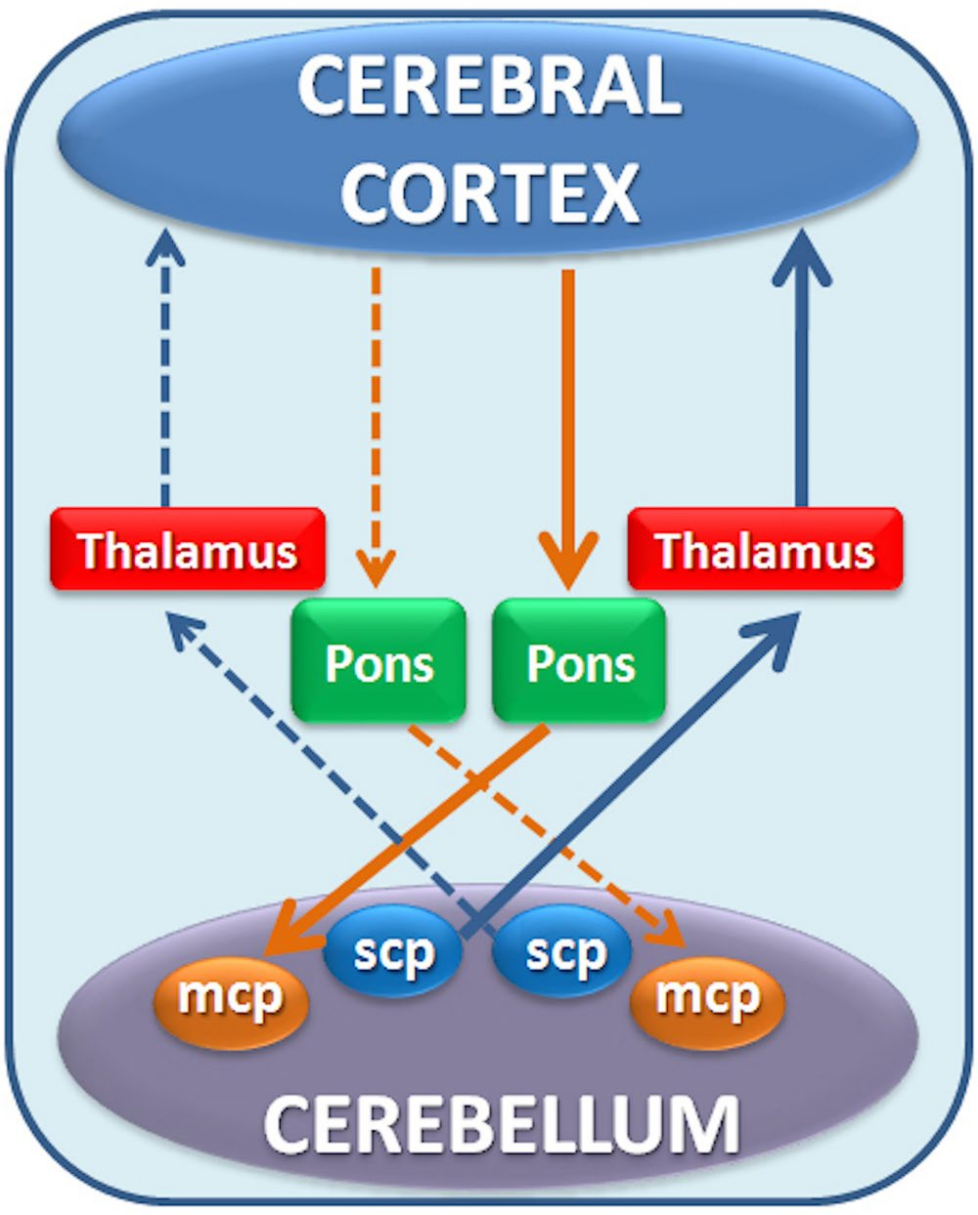
Schematic diagram of the cerebro-cerebellar loop. The cortico-ponto-cerebellar pathway (orange arrows) connects the cerebrum with the cerebellum passing through the pons and the contralateral middle cerebellar peduncle (MCP). The cerebello-thalamo-cortical pathway (blue arrows) connects the cerebellum with the cerebrum passing through the superior cerebellar peduncle (SCP) and the contralateral thalamus. Dotted arrows represent the contralateral pathway with corresponding colours.

Increasing evidence implicates the cerebellum both in motor control^1^ and in cognitive processing^2^. The cerebellum exerts its functions in close communication with the cerebral cortex by exploiting two main pathways: the efferent cerebello-thalamo-cortical (CTC) pathway and the afferent cortico-ponto-cerebellar (CPC) pathway (Fig. 1)^3–5^.

The anatomy of these pathways has been partially described in humans *in vivo* ^6–8^; in detail, the CTC pathway has been characterized in our previous investigation^9,10^, hence the present study focuses on reconstructing the CPC pathway. New understanding of the cerebellar connectivity in humans *in vivo* would provide essential information for determining the pathophysiological mechanisms involved in a number of clinical conditions. Indeed, an increasing number of investigations suggest that the abnormal functioning of these loops may subtend major neurological conditions including dystonia, ataxia, hemiplegia, stroke and autism^11–15^. Furthermore, characterizing the cerebro-cerebellar loops quantitatively would be extremely important for computational modelling of this loop.

Several investigations *ex vivo* have provided information on the anatomical nature of these pathways, whereas less is known in humans *in vivo*. From *ex vivo* studies it is possible to assert that the CTC pathway originates from the cerebellum and, passing through the superior cerebellar peduncle, reaches the contralateral cerebral cortex via synapses in the contralateral ventro-anterior and ventro-lateral thalamic nuclei^3,16^; while the CPC pathway originates from the cerebral cortex and, after descending through the ipsilateral cerebral peduncle (CP), synapses in the anterior pontine nuclei (APN) and passes through the contralateral middle cerebellar peduncle (MCP) before reaching the cerebellar cortex^4,17^.

The CPC pathway has been examined by tracing techniques in experimental animals^4,17^, showing projections to the pons from several cerebral areas^3,18–20^. For example, Kelly and Strick^17^ reported trans-synaptic pathways traveling from primary motor (M1) and prefrontal cortices to distinct locations in the cerebellar cortex, mainly the lobules V-VI and Crus II respectively, in the primate brain, while Schmahmann and Pandya demonstrated connections between the superior temporal lobe and the pontine nuclei^19^. Furthermore the CTC and CPC pathways have been described in humans *ex vivo* using Nissl staining in post-mortem brains after leucotomy^21^ and using confocal laser and polarized light microscopy^22^. All these studies are focusing on specific aspects of the cerebro-cerebellar loop and support the knowledge that there is a complex interaction between cerebellum and cerebral cortex, involving both motor and non-motor areas.

Previous MRI studies^23–27^ have indeed assessed the existence of a convincing relationship between the CPC pathway and cognition in humans *in vivo*. One approach has been to rely on functional MRI (fMRI) results to first identify cerebral and cerebellar regions that are functionally connected, then to examine structural connections between those pre-defined regions^23–25^. Tractography based on diffusion weighted imaging^28^, despite its limitations (e.g. impossibility of detecting synapses, resolving complex tissues microstructures and inclusion of false positive results)^29,30^, is currently the only method to investigate important characteristics of specific neuronal pathways in humans *in vivo*. Previous studies using deterministic tractography based on the diffusion tensor model have assessed the feasibility of reconstructing these pathways *in vivo*. In particular, Kamali, *et al*.^7^ reconstructed different portions of the CPC pathway, e.g. fronto-ponto-cerebellar and temporo-ponto-cerebellar tracts, while Keser, *et al*.^8^ described both CTC and CPC pathways in terms of their volumes and diffusion parameters along them. The reported findings, though, were partially inconsistent with previous neuroanatomical investigations^4,17^. This can be explained due to their use of diffusion-tensor based tractography, which cannot resolve crossing fibres, meaning that the reconstructed tracts were ipsilateral with the exception of the fronto-ponto-cerebellar tract.

The aim of this study is therefore to reconstruct the contralateral CPC pathway with the same pipeline used for the CTC pathway in order to gather critical information about the cerebro-cerebellar loops involving motor and non-motor areas. In particular, the study aims to assess whether: *(1)* the contralateral CPC pathway can be reconstructed by simply constraining streamlines to pass through the MCP and the contralateral CP; *(2)* there is a correspondence between cerebral and cerebellar cognitive areas via the MCP, and how that supports the results found for the CTC pathway; *(3)* it is possible to hypothesize the existence of cerebro-cerebellar closed-loops by comparing the areas belonging to the CPC and the CTC pathways. Here, in order to achieve these aims, the reconstruction of the CPC pathway was accomplished by combining CSD, that models multiple fiber populations within a voxel resolving the decussation of trans-hemispheric connections, with TDI, that provides maps with higher resolution and contrast than with classical diffusion tensor imaging derived maps (e.g. fractional anisotropy, and mean diffusivity), which are useful to place seed and target regions of interest (ROIs)^31^. Moreover, to quantify the pattern of correspondence of cerebro-cerebellar cortical regions reached by the computed CPC pathway and to assess the relationship between areas associated to cognitive and motor functions, two previously defined metrics were used^9^: trGM_cROI_ to assess the proportion of the tracts projecting to a particular ROI and cROI_tract_ to assess the proportion of a given ROI involved in the tract. For completeness, the raw total streamlines count (TSC) for each cortical region was also reported.

## Results

### The reconstruction of the CPC pathway

**Figure 2:**
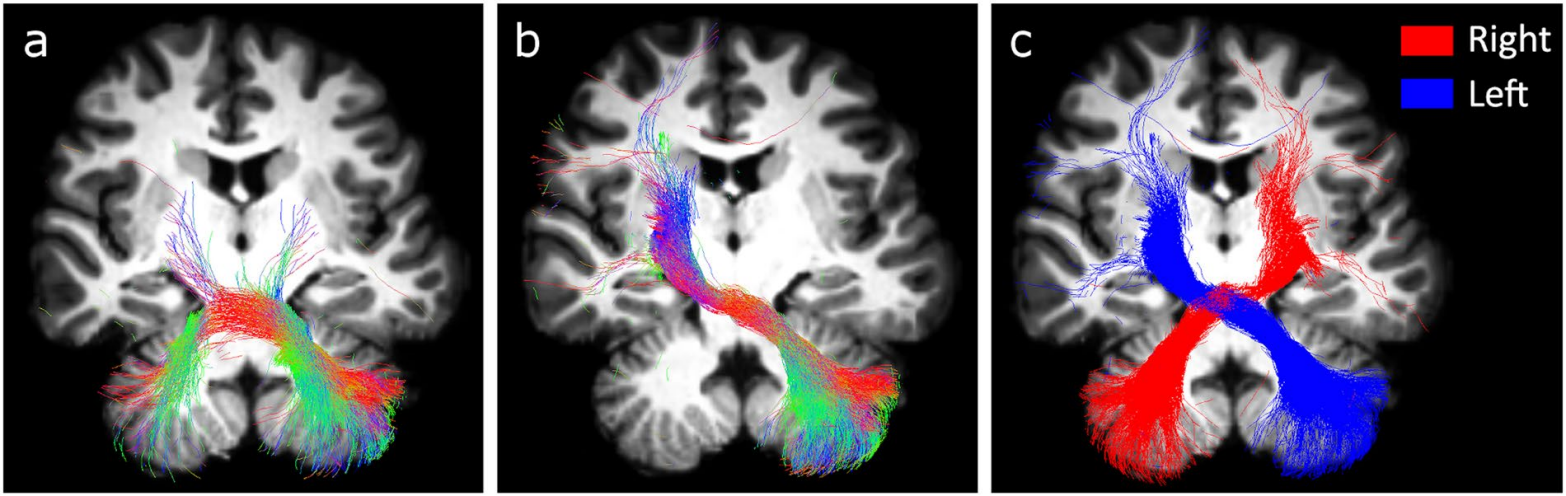
2D rendering of combined CSD technique and probabilistic streamlines tractography in a representative subject. Streamlines in (**a)** and (**b**) are colour-coded according to the diffusion-derived fiber direction, while in (**c**) two solid colours differentiate the left and the right seeded pathways. (**a**) Pathway reconstructed with a seed ROI placed in the left middle cerebellar peduncle. No target ROI was used. (**b**) Pathway reconstructed with a seed ROI in the left middle cerebellar peduncle and a target ROI in the contralateral cerebral peduncle. (**c**) Pathways reconstructed as in (**b**) using both left (blue) and right (red) seed ROIs.

Both the left and right seeded contralateral CPC pathways were successfully reconstructed in all subjects by combining CSD and probabilistic tractography. Figure 2 shows an example from a representative subject. As shown in Fig. 2a using a seed in the MCP without any other constraint, the algorithm reconstructs both ipsilateral and contralateral tracts as well as streamlines that run from one cerebellar hemisphere to the opposite one. When adding appropriate target ROIs, the algorithm reconstructs only tracts contralateral to the MCP (Fig. 2b,c).

**Figure 3:**
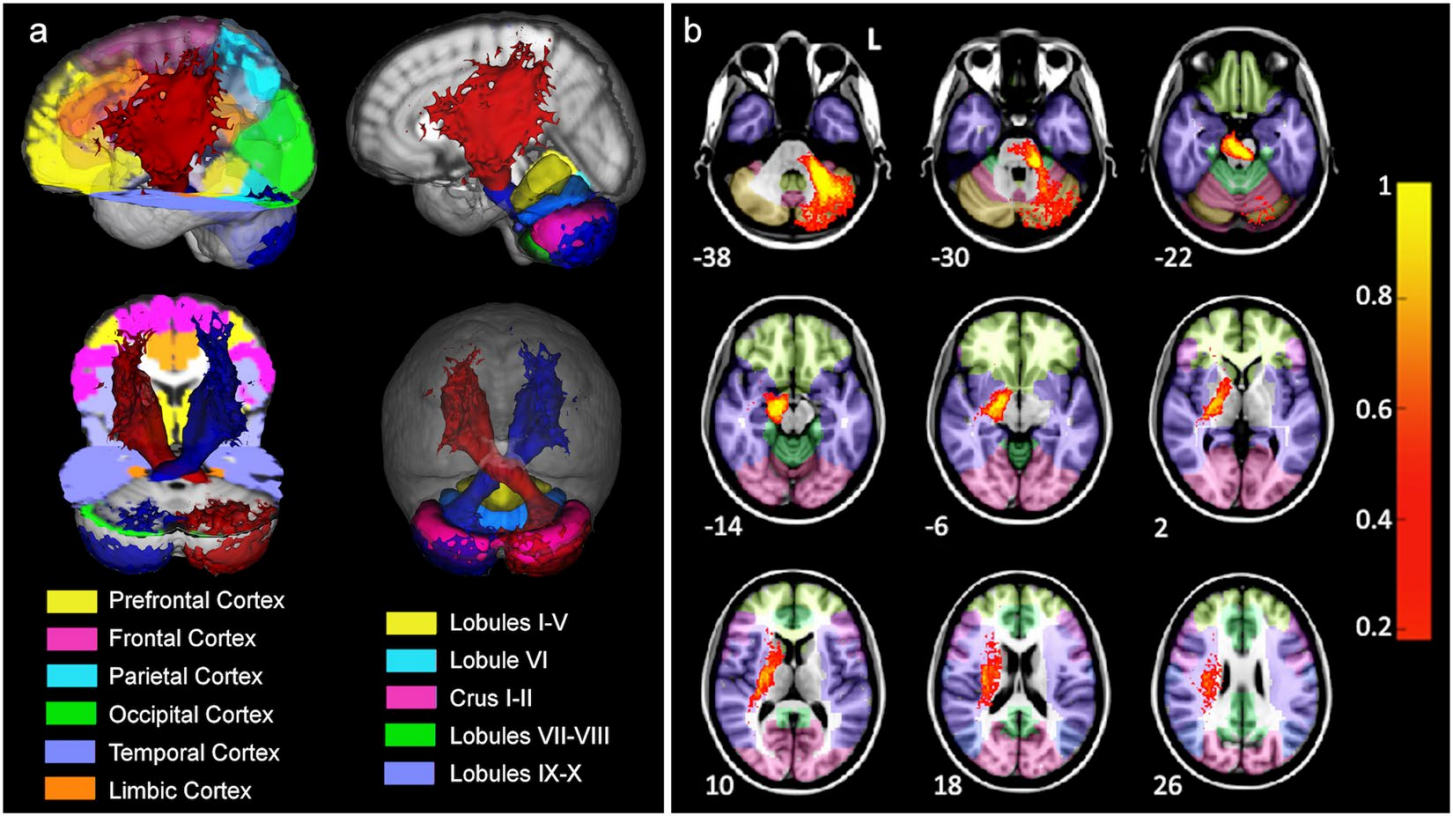
Extension of the cortico-ponto-cerebellar pathways averaged across all subjects, normalised to MNI space and thresholded to include voxels common to at least 20% of subjects. (**a**) Tridimensional view overlaid on cerebral (left panel) and cerebellar (right panel) anatomical parcellations. Temporal (violet) and frontal (pink) lobes are the areas mainly reached both by the left (blue) and right (red) seeded pathways. In the cerebellum, lateral crus I-II (pink) and lobules VII-VIII (green) are areas with the greatest density of streamlines. (**b**) Axial sections of the left pathway (red-yellow) overlaid on the same anatomical templates. Z coordinates are reported for each slice (mm). L = left side of the brain. The scale on the right represents the mean pathway coloured in terms of percentage of overlapping subjects (1 = 100%).

**Figure 4:**
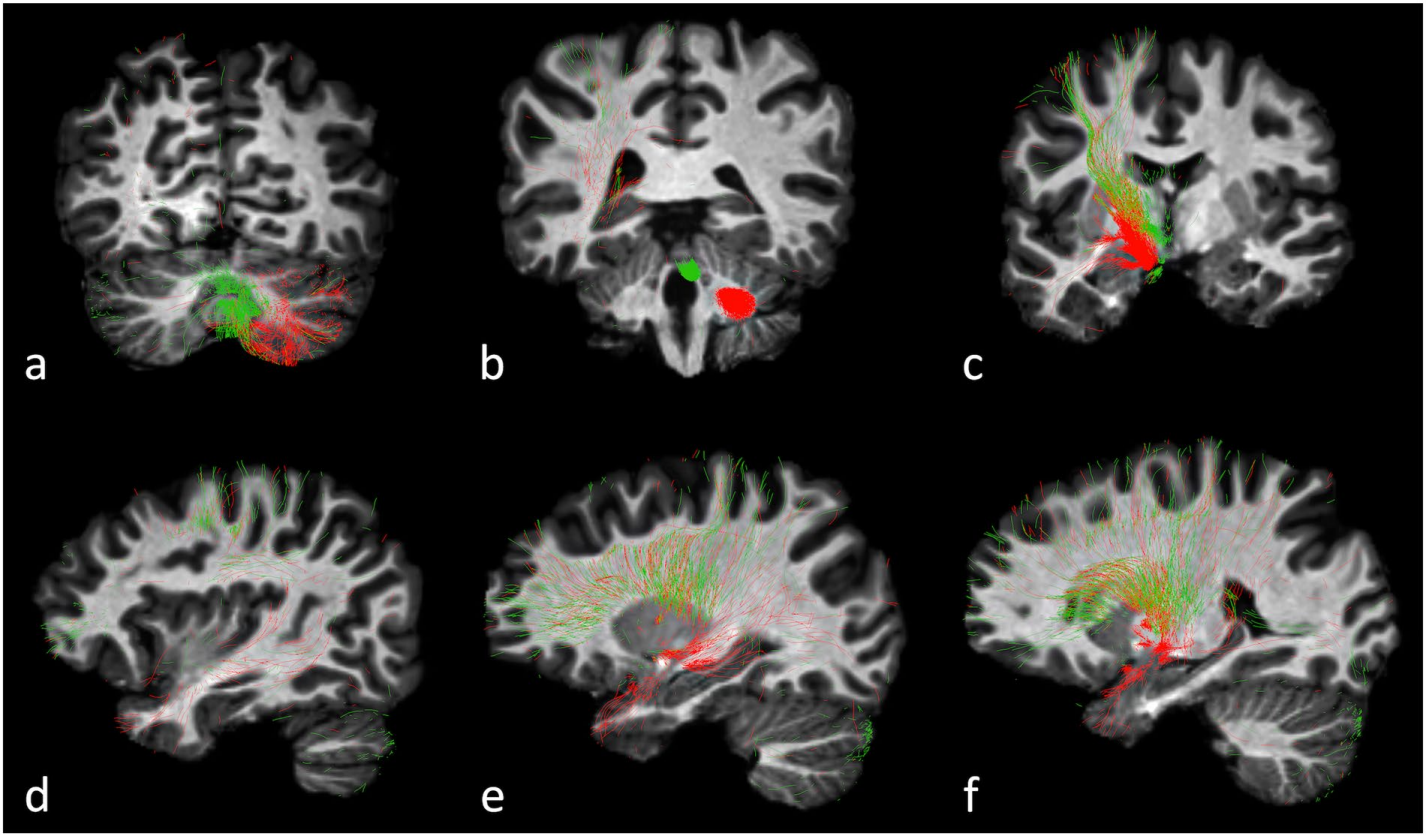
Left seeded cortico-ponto-cerebellar tract (red) and cerebello-thalamo-cortical tract (green) of a representative subject. In the cerebellum both pathways occupy mainly the lateral crus I-II and lobules VIIb/VIII (**a**), while outputs from the cerebellum (**b**) are different. In cerebral areas they partially overlap in the frontal lobe (**b**,**d**,**e**). The temporal lobe, instead, is reached more densely by the CPC streamlines than the CTC ones, while in the prefrontal cortex there are more CTC than CPC streamlines (**d**,**e**,**f**).

In order to highlight the main skeleton of the CPC pathways, Fig. 3a shows the tridimensional mean pathway across all subjects normalized to MNI space and thresholded to include voxels that are common to at least 20% of subjects, while Fig. 3b shows sections of the same mean pathway (only left seeded for clarity) overlaid on the MNI-152 anatomical template. Moreover, Fig. 4 shows the streamlines of the CPC pathway (red) in a representative subject to demonstrate the whole extent of the CPC pathway and to consistently identify its different portions, e.g. fronto-ponto-cerebellar or temporo-ponto-cerebellar tracts. It is worth noting that areas reached by the majority of streamlines are the temporal and frontal lobes (BA 4, 6, 8, 44, 45) in the cerebrum, and the lateral Crus I-II and lobules VIIb/VIII in the cerebellum. Moreover, the CPC pathway does not enter the thalamus but instead runs just outside this area, passing correctly through the internal capsule (see Fig. 3b: 2 and 10 mm MNI slices)^18^.

### Tractography metric

Here are reported results (trGM_cROI_, cROI_tract_ and TSC values), based on anatomical and functional parcellations^32–34^.

#### Anatomical parcellations

In Table 1 trGM_cROI_, cROI_tract_ and TSC values are reported for both cerebral and cerebellar parcellations. In the cerebrum, the three metrics have the highest values in the temporal lobe. In the cerebellum, the metrics have the highest values in the lateral Crus I-II.

**Table 1:** trGM_cROI_, cROI_tract_ and TSC values of cerebral and cerebellar cortical areas defined on anatomical bases.

Structure	Anatomical Areas	*trGM*_*cROI*_ *(SD) (%)*	*cROI*_*tract*_ *(SD) (%)*	*TSC (SD)*
**Cerebrum**	Prefrontal Cortex	8(5)	1.1(0.6)	352(208)
	Frontal Lobe	20(6)	3.5(1.2)	888(269)
	Parietal Lobe	9(4)	1.9(1.3)	494(285)
	Temporal Lobe	**54(6)**	**3.8(1)**	**3355(816)**
	Occipital Lobe	6(2)	1.1(0.6)	446(160)
	Limbic Lobe	3(1)	1.1(0.4)	723(392)
**Cerebellum**	Anterior Lobule (I-V)	2(1)	2.5(1)	1017(515)
	Lobule VI	11(3)	15.2(4.1)	1420(327)
	Lateral Crus I-II	**62(4)**	**34.9(2.5)**	**4336(219)**
	Lobules VIIb/VIII	24(5)	22(5.8)	2286(450)
	Inferior Lobule(IX-X)	1(0)	2.3(1.3)	219(174)

Each value is averaged over subjects. Data are expressed as mean (SD) for each area. Highest values are bold labelled. trGM_cROI_ = GM tract volume in one cortical area relative to GM tract volume in all cortical areas; cROI_tract_ = GM tract volume in one cortical area relative to the area volume itself; TSC = total streamline count. Note: the TSC value may exceed 3000 streamlines because, for each area, it is obtained averaging over subjects the results calculated adding left and right seeded streamlines together.

A careful inspection of Table 1 reveals that the main findings are:

- Overall, in the cerebellum the posterior-lateral areas (Crus I-II and lobules VIIb/VIII) are the ones most reached by the tract.
- The range of trGM_cROI_ values is similar between the lateral Crus I-II (62% ± 4%) and the temporal lobe (54% ± 6%).
- Lower percentages of trGM_cROI_ involved the lobules I-VI (13% ± 4%) and VIIb/VIII (24% ± 5%) in the cerebellum and the frontal lobe (20% ± 6%) and prefrontal cortex (8% ± 5%) in the cerebrum.

#### Functional parcellations

In Table 2 trGM_cROI_, cROI_tract_ and TSC values are reported for both cerebral and cerebellar parcellations. In the cerebrum, the highest trGM_cROI_ and TSC are found in the associative areas, while cROI_tract_ has the highest value in the motor areas. In the cerebellum, the metrics have the highest values in the cognitive/sensory area.

**Table 2:** trGM_cROI_, cROI_tract_ and TSC values of cerebral and cerebellar cortical areas defined on functional bases.

Structure	Functional Areas	*trGM*_*cROI*_ *(SD) (%)*	*cROI*_*tract*_ *(SD) (%)*	*TSC(SD)*
**Cerebrum**	Motor Area	19(6)	**4.3(1.5)**	844(268)
	Associative Area	**69(7)**	2.5(0.6)	**3865(864)**
	Primary Somato-sensory	4(2)	3.4(2.1)	267(154)
	Primary Visual Area	6(2)	1.1(0.6)	446(160)
	Primary Auditory Area	2(1)	3.2(2.9)	119(97)
**Cerebellum**	Primary motor area	2(1)	2.5(1)	1017(515)
	Cognitive/Sensory Area	**96(1)**	**25.6(2.3)**	**5858(210)**
	Sensory-Motor Area	2(1)	11.4(3.3)	354(115)

Each value is averaged over subjects. Data are expressed as mean (SD) for each area. Highest values are bold labelled. trGM_cROI_ = GM tract volume in one cortical area relative to GM tract volume in all cortical areas; cROI_tract_ = GM tract volume in one cortical area relative to the area volume itself; TSC = total streamline count. Note: the TSC value exceeds 3000 streamlines because, for each parcellation, it is obtained averaging over subjects the results calculated adding left and right seeded streamlines together.

A careful inspection of Table 2 reveals that the main findings are:

- In the cerebellum almost all streamlines reach the cognitive/sensory area (trGM_cROI_ = 96% ± 1%).
- The associative areas of the cerebrum encompassed the highest percentage of streamlines (trGM_cROI_ = 69% ± 7%).
- Consequently about 70% of the pathway is likely to involve cerebral and cerebellar cognitive areas.

## Discussion

This work has successfully reconstructed the contralateral CPC pathway and has actively contributed to the open discussion on *in vivo* properties of the cerebro-cerebellar loops. Indeed, together with the same quantitative metrics previously calculated for the CTC pathway, our results support the coevolution of two cortices (cerebral and cerebellar) previously proposed on the basis of comparative cortical surface measurement across vertebrates^35^, and their structural connectivity forming multiple closed loops involving not only motor but also cognitive areas. It is worth noting that our findings are the first CSD-based tractography reconstructions that give evidence of a complex system of cerebro-cerebellar closed loops going beyond the pure motor system.

One key novel aspect of our work, compared to the previous contributions of Kamali, *et al*.^7^ and Keser, *et al*.^8^, is to employ advanced probabilistic tractography and track-density imaging (TDI) combined with constrained spherical deconvolution (CSD)^36^, which models multiple fiber populations within a voxel and resolves crossing fibres issues. Moreover, in this work we do not impose cortical parcellations prior to running the tractography, differently from previous studies^7,8^, hence our analysis is not limited to specific cortical regions (e.g. motor), while at the same time ensuring a high degree of fidelity to known anatomy by imposing some a priori key conditions. Indeed, according to anatomical knowledge by using the superior cerebellar peduncle as tractography seed region and the red nucleus as waypoint to constrain the pathway of the streamlines, it was possible to reconstruct an unconstrained distribution of target cortical regions reached by efferent streamlines originating from the cerebellum.

To quantify the pattern of correspondence of cerebro-cerebellar cortical regions reached by the computed CPC pathway and to assess the relationship between areas associated to cognitive and motor functions, two previously defined metrics were used^9^: trGM_cROI_ to assess the proportion of the tracts projecting to a particular ROI and cROI_tract_ to assess the proportion of a given ROI involved in the tract. For completeness, the raw total streamline count (TSC) for each cortical region has also been reported for the CPC as for the CTC pathways. These metrics helped us to assess the potential correspondence between the cerebral cortex and the cerebellum, compare results of the cerebellar CPC afferent pathway with those of the efferent CTC one, in an attempt to define the complexity of the cerebro-cerebellar loops. Furthermore, it is worth noticing that the reconstructed pathways are more extended than those provided in previous diffusion-tensor based works^7,8^, as CSD facilitates tracts to reach within cortical areas.

Current neuroanatomical knowledge anticipates that the CPC pathway starts from cerebral cortical areas, passes through the CP, reaches the contralateral MCP as a coherent bundle, and finally spreads towards the cerebellar cortex^3,4^. This organization poses a key problem for diffusion-tensor-based tractography, that of following crossing fibers. Thus, in this work a non-tensor model combining CSD algorithm with probabilistic tractography^13,14,37^ was adopted, as already successfully done for the CTC pathway reconstruction^9^. Some fidelity to the known anatomical characteristics of the CPC pathway was imposed by using a relatively unconstrained approach (ROIs were placed in the MCP and in the CP, allowing streamlines to freely reach any cortical areas), and the resulting cerebral and cerebellar areas involved in the reconstructed pathway were in agreement with the anatomical findings of *ex vivo* experiments^4,17,19^. Moreover, three previously reported indices were used to quantify the results^9^, and thus allow direct comparison to previous findings: trGM_cROI_ to assess the proportion of the tracts projecting to a particular ROI, cROI_tract_ to assess the proportion of a given ROI involved in the tract, and TSC to reflect the raw number of the streamlines reaching a specific cortex. Using these indices, it was possible to infer the correspondence of involvement between specific cerebral and cerebellar areas. Of course, the specific contribution of different cortical regions to the cerebro-cerebellar loop remains to be carefully evaluated considering the implications linked to the chosen cortical parcellation atlas and the effective ability of diffusion tractography to provide quantitative comparative information on long-range transynaptic pathways, as further discussed below.

Despite the intrinsic limitations of diffusion tractography, the results of this study are in line with previous findings using different techniques. Indeed, both *ex vivo* and *in vivo* studies have reported that the majority of frontal lobe connections involves lobules V-VI while fewer connections involve lobules VIIb/VIII^4,17,26,27^. In the present study the frontal lobe, which comprises the motor areas (BA 4, 6) as well as the frontal eye field (BA 8) and Broca’s areas (BA 44-45), accounts for 20% of the CPC pathway, showing the second highest trGM_cROI_ value in the cerebrum, while lobules I-VI account for 13% of the CPC pathway. Thus, we hypothesize that trGM_cROI_ of the frontal lobe (20% ± 6%) finds cerebellar correlate in lobules I-VI (13% ± 4%) and only partially in lobules VIIb/VIII (24% ± 5%). Moreover, this hypothesis could explain the discrepancy between trGM_cROI_ values of the cerebral motor (comprising both premotor cortex and M1) and the cerebellar primary motor (lobules I-V) areas (19% ± 6% and 2% ± 6% respectively). The high number of streamlines involving the frontal lobe is in agreement with viral tracing experiments where BA 4, 6, 8 project a large amount of fibers to the pons^18^ and then to the cerebellum^4,17^, but also find correspondence in several fMRI studies which found coherent activity between the primary motor cortex and lobules V, VI and VIII^38,39^. Furthermore, cerebellar lobule VII has been shown to activate during cognitive tasks and coherent functional connectivity has been found between lobules VIIb/VIII and frontal lobe in observation learning^5,39,40^. Altogether these considerations support our findings of streamlines between the frontal lobe and the cerebellum and, therefore, the cerebellar involvement in sensorimotor control and related cognitive functions.

Moreover, several tracing and MRI studies have provided evidence of connections between other cerebral areas, such as parietal, prefrontal and temporal cortex, and the cerebellum. In particular, a number of studies mapped the bidirectional connection between the parietal lobe and the cerebellum^3,41,42^ using viral tracing techniques in animals while fMRI studies have revealed coherent functional connectivity between these regions^43^. Here is reported that the parietal lobe encompasses 9% of the CPC pathway. Since the parietal lobe is known to be involved in response to the sight of an object, as well as to the act of grasping it^44^, and in the creation of cross-modal sensorial representations of objects^45^, results of the present study support the cerebellar involvement in spatial orientation and representation functions. Furthermore, tracing experiments in non-human primates^17,20^ have identified a “prefronto-cerebellar bundle” connecting the prefrontal cortex mainly with the Crus I-II, which has also been found by functional and structural studies in humans *in vivo* ^26,39,46,47^. In line with such findings, the current results confirm that about 8% of the CPC streamlines leaves the prefrontal cortex (BA 9-12, 25, 46–47), supporting the cerebellar role in cognitive functions, such as working memory and mental preparation for imminent actions. Lastly, the contribution of temporal fibers to the CPC pathway has been demonstrated with experiments on rhesus monkeys^3,19^, whereas recent studies using fMRI^25,47–49^, tractography^24^ and dynamic causal modelling^50^ have reported a strong bidirectional connection between the superior temporal sulcus and the Crus I-II in humans. The results here show a coincidence of trGM_cROI_ values between the temporal lobe (as parcellated by Brodmann and including the hippocampus and amygdala)^34^ and Crus I-II (54% ± 6% and 62% ± 4% respectively), suggesting that the two areas could be interconnected trough the CPC pathway^5,11^ and supporting the involvement of Crus I-II and the temporal lobe in multimodal cognitive functions^5,25,39^. This study reports a greater amount of streamlines leaving the temporal lobe compared to those expected from previous studies that employed probabilistic or deterministic tractography based on *in vivo* diffusion tensor imaging in humans and macaques^7,8,26^. Other than these, there are no other reported quantifications of the temporo-cerebellar connectivity *in vivo* to compare results with. The greater amount of temporo-ponto-cerebellar connections found in our study can be explained by the use of a non-tensor model for tractography and a different cerebral cortical parcellation. To support our findings, we have noticed and reported that previous tract tracing studies have given evidence of temporo-ponto-cerebellar tracts from the superior temporal sulcus (e.g. Schmahmann 1997), but literature about which tract is greater among those from temporal, parietal and occipital lobes is lacking and controversial^3,19,26^.

Overall, all these findings support that the cerebellum has indeed an important role in cognition as the prefrontal cortex (BA 9–12, 25, 46–47), language areas (BA 44–45), parietal (except BA 1–3), temporal (except BA 41–42) and the limbic lobes all together encompass 69% of the tract in the cerebrum, while cerebellar cognitive hemispheres encompass 96% of the tract^33,39,47^. As far as sensorimotor areas are concerned with, the cerebral motor area (comprising BA 4, 6, 8) and the anterior part of the cerebellum (lobules I–VI) show a high involvement (trGM_cROI_ is 19% ± 6%), in agreement with the cerebellar role in motor functions. In contrast, the primary sensory cortices account for just a minor fraction of the CPC streamlines (primary auditory and visual cortices are 2% and 6%, respectively). These values are in accordance with fMRI studies showing that primary auditory and visual cortices do not appear functionally coupled with the cerebellum^43^. However, a functional relationship of these cortices with the cerebellum should exists, since connections from the fastigial and vestibular nuclei passing through the inferior and the middle cerebellar peduncles play an important role in controlling the execution of saccades and in elaborating the visuospatial information concerning the eye target^51^. An underestimation by tractography methods of these connections has to be considered: it could be caused by the high curvature of fibers connecting the cerebellum with visual and auditory areas (located in the occipital and inferior part of the temporal lobes, respectively). The cerebrum somato-sensory area constitutes only 4% of the CPC pathway. Since works based on functional connectivity reported an existing network between the cerebellum and this cerebral area^38,43^, further studies with new analysis methods^52,53^ could help to clarify the issue.

Beyond the characterisation of the CPC pathway itself, it is fundamental to combine the present results with those of previous work on the CTC pathway performed with identical tractography pipeline (apart from seed and target regions)^9^ to improve knowledge about the cerebro-cerebellar closed-loops. Indeed, the cerebro-cerebellar connections are thought to form closed-loops, with the feedforward and feedback branches passing mainly through the CPC and CTC pathways, respectively^3–5^. Thanks to these loops, the cerebellum is thought to operate as an information processing system that modulates cerebral activity^2,4^. For example, the error-correction model posits that the cerebellum behaves as an internal simulator, which receives inputs from the cortex via the CPC pathway and modulates cerebral processing via the CTC pathway^54,55^. These loops might be closed either by direct connections of the same cerebral areas or pass through intra-cerebral connections both concerning sensorimotor and associative intermediate loops^56^. As far as the sensorimotor areas are concerned, in the frontal lobe (comprising BA 4, 6, 8) and in the lobules I–VI, CTC and CPC pathways showed a similar range of trGM_cROI_ values. Therefore, despite all tractography caveats^29,57^ (discussed further on), the symmetry of CTC-CPC pathways in motor areas would support a cerebro-cerebellar closed-loop for controlling motor programming and execution^1^. Conversely, about 70% of the CPC pathways were described as connecting associative areas of the cerebral cortex to the contralateral cerebellar cognitive areas, in a proportion almost equating the ratio reported for the CTC pathways (80%)^9^. Despite this analogy, our results showed that the CTC pathways reached mainly the prefrontal cortex (trGM_cROI_ 38% ± 11%) and the temporal lobe (trGM_cROI_ 35% ± 5%), while the CPC pathways originated preferentially from the temporal lobe (trGM_cROI_ 54% ± 6%) rather than from prefrontal cortex (trGM_cROI_ 8% ± 5%). The discrepancy found in the amount of CTC streamlines reaching the prefrontal cortex^9^, compared with CPC streamlines leaving it, may reflect a complex network structure, rather than being simply due to tractography limitations. Indeed, it is well known that several tracts exist, which are connecting sensory areas in the occipito-parietal, temporal lobes and the frontal lobe, including the arcuate fasciculus connecting the Wernicke to the Broca areas^58^. As far as the prefrontal cortex and temporal lobe are concerned with, histological and MRI studies have shown that the uncinate fasciculus bidirectionally connects the prefrontal cortex and the temporal lobe^58,59^, whereas these areas are also probably connected through the fornix and the parahippocampal cingulum^60,61^. Therefore, current results provide the first quantitative MRI-based evidence of *in vivo* cerebro-cerebellar connectivity that, if confirmed, would be useful for informing closed-loop models for both sensorimotor and cognitive control functions by providing the proportions of CTC and CPC streamlines that are reaching different cerebellar and cerebral cortices^55,62^. CTC and CPC pathways of a representative subject are reported in Fig. 4 in order to provide a visual evidence of the mutual connectivity between them.

The intrinsic limitations typical of MRI tractography also apply to the present study (see also Palesi, *et al*.^9^ for further discussion); nevertheless, tractography is the only available method for reconstructing axonal bundles in humans *in vivo*. In general, MRI tractography cannot distinguish between efferent and afferent tracts, cannot discriminate between mono-synaptic and trans-synaptic connections, cannot resolve single axonal fibers, and is known to produce large number of false-positive streamlines^29,30,57^. Nevertheless, the common issue of resolving crossing fibres was addressed here by employing an algorithm that models multiple fibre populations within a voxel^37^. Here below, technical considerations with respect to CPC reconstruction and the adopted pipeline are discussed with further detail.

Firstly, the fact that MRI tractography cannot distinguish between efferent and afferent tracts implies that the direction of axon potential propagation has to be inferred using *a priori* knowledge. Indeed, we placed seed/target ROIs on the basis of findings from anatomical studies^17,18^ and on the basis of previous tractography studies^7,13^ demonstrating that the streamlines passing through the CP and the MCP can be identified as part of the major bundle unidirectionally connecting the cerebral cortex to the cerebellum. Despite of, the reconstructed streamlines, “upstream” of CP and “downstream” of MCP, were allowed to run unconstrained towards the cerebral and cerebellar cortices, respectively.

Secondly, MRI tractography is not able to discriminate between mono-synaptic and trans-synaptic connections because the diffusion weighted signal is influenced by the average microstructural architecture over the scale of an imaging voxel and not by the presence/absence of the synapses. In the CPC pathway, pontine nuclei are synaptic relays receiving descending fibers from the CP and sending them into the cerebellum almost uniquely through the MCP, contralaterally. Interestingly, using this anatomical information as constrain, the pipeline used in this study (combining CSD and TDI) was able to follow the fibers curvature and their trans-synaptic collaterals in the pons.

Thirdly, diffusion MRI data have poor resolution (mm scale) compared to the scale of single axonal fiber (μm scale) and may not be able to robustly follow axonal bundles at crossing points. Moreover, the extent to which specific tracts might be lost depending on the presence of synapses or axonal bending or fiber crossing in the complex situation of *in vivo* tissue remains unknown. Quantifying the false positives of fiber tracking using *in silico* phantoms have claimed a high percentage of spurious streamlines^30^. How this proportion can be reduced by imposing *a priori* knowledge, like in the present study, has to be verified. Thus, in general, neither the absolute fiber number or density, nor the strength of connections can be precisely determined with tractography^57^.

Finally, beyond a reliable identification of the CPC pathways bilaterally, there is the problem of evaluating to what extent the present analysis can provide a quantitative description of the involved tracts. The issue is particularly relevant since the proportion of CPC and CTC tracts connected to the same cortical areas were asymmetric in some cases, and since some connections (like that with the temporal lobe) seemed to be over-represented compared to results reported by previous works^4^. It is worth noting that these discrepancies between present results and those reported by previous studies^7,8,26,27^ may strongly depend upon the different cortical parcellation that has been chosen (here cortical parcellations as defined by Brodmann^34^ were used) and upon the mathematical model and tractography algorithm that have been used. Although no reliable conclusion on tract size can be drawn at present, we tried to limit this problem by using the same MRI scans and the same analysis pipeline in both the CTC and CPC pathways reconstructions, so that direct comparisons of the CPC and CTC quantitative metrics would be performed^9,10^. Further improvements may be achieved by exploiting recent developments in diffusion MRI tractography methods^52,53^ and by repeating the analysis using higher resolution data, such as those available through the Human Connectome Project (www.humanconnectomeproject.org). Ultimately, unless moving to tracers studies in animals or post mortem studies in humans, tractography is currently the only available technique for the investigation of complex pathways in humans *in vivo*, therefore it is important that studies such as this one are performed and discussed, taking into consideration a comprehensive knowledge gathered from multiple modalities, anatomical studies and non-human primate and animal investigations.

## Conclusions

The work reported in this study led to the characterization of the descending pathway connecting the cerebral cortex with the contralateral cerebellar hemisphere, passing through the CP and the contralateral MCP in humans *in vivo*. Evidence of congruent streamline metrics of the cerebral and cerebellar cortices in cognitive areas bears relevant functional implications supporting the existence of a complex network with multiple closed-loops, which comprises also intra-cortical cerebral connectivity. This result confirms the coevolution of the two cortices proposed on the basis of comparative cortical surface measurement across vertebrates^35^. Since the cerebellar cortex has almost an identical structure in all its sections and is organized in parallel poorly-interacting modules^3^, it is possible that a similar computational algorithm is applied to different cortical functions, ranging from motor control to sensory perception and cognition^11^, supported by the presence of pathways connecting different cerebral and cerebellar cortices (CTC and CPC). Quantification of the involvement of different cerebral areas, depending on their motor or cognitive function, would provide very relevant information for the development of computational models of cerebro-cerebellar loops^55^. Since the analysis was performed on high-angular resolution diffusion imaging (HARDI) data acquired on a standard clinical scanner, this method could be applied in the assessment of the integrity of the cerebro-cerebellar connectivity in pathologies (e.g. dystonia, ataxia and autism) for which a cerebellar involvement has been proposed^11,12,14^.

## Material and Methods

### Subjects

To directly compare CTC and CPC pathways results, the same cohort of subjects as in Palesi, *et al*.^9,10^ was analyzed: 15 right-handed healthy adults (7 males and 8 females; mean age 36.1 years and range 22–64 years) with no previous history of neurological symptoms. All participants gave written informed consent for the study and for the publication of research data and images. The study protocol was approved by the local institutional research ethics committee (NRES Committee London-Queen Square) and all experiments were performed in accordance with relevant guidelines and regulations.

### MRI acquisition

All data were acquired on a Philips Achieva 3 T MRI scanner (Philips Healthcare, Best, The Netherlands) using a 32-channel head coil. The HARDI scan consisted of a cardiac-gated SE echo-planar imaging (EPI) sequence acquired axial-oblique and aligned with the anterior commissure/posterior commissure line, for a total scan time of approximately 20 min. The imaging parameters were TR ≈ 24 s (depending on the cardiac rate), TE = 68 ms, SENSE factor = 3.1, acquisition matrix = 96 × 112, 2 mm isotropic voxel and 72 axial slices with no gap. The diffusion weighting was distributed along 61 optimized non-collinear directions with a b value of 1,200 s/mm^2,63^. For each set of diffusion-weighted data, 7 volumes with no diffusion weighting (b = 0) were acquired. For anatomical reference a whole brain high-resolution 3D sagittal T1-weighted (3DT1w) fast field echo (FFE) scan was acquired using the following parameters: TR = 6.9 ms, TE = 3.1 ms, TI = 824 ms, acquisition matrix = 256 × 256, 1 mm isotropic voxel, 180 sagittal slices, acquisition time 6 min 31 s.

### Diffusion analysis and fiber tracking

HARDI data were analysed using FSL (FMRIB Software Library, http://fsl.fmrib.ox.ac.uk/fsl/fslwiki/)^64^ and MRtrix (http://www.brain.org.au/software/mrtrix/)^37^ software packages. Both pipeline and parameters used for the analysis were described in detail in Palesi, *et al*.^9^; a brief summary of the main steps will follow (steps 3, 8 and 15 of Palesi, *et al*.^9^ will not be reported here as they are not pertinent to this work).

**Figure 5:**
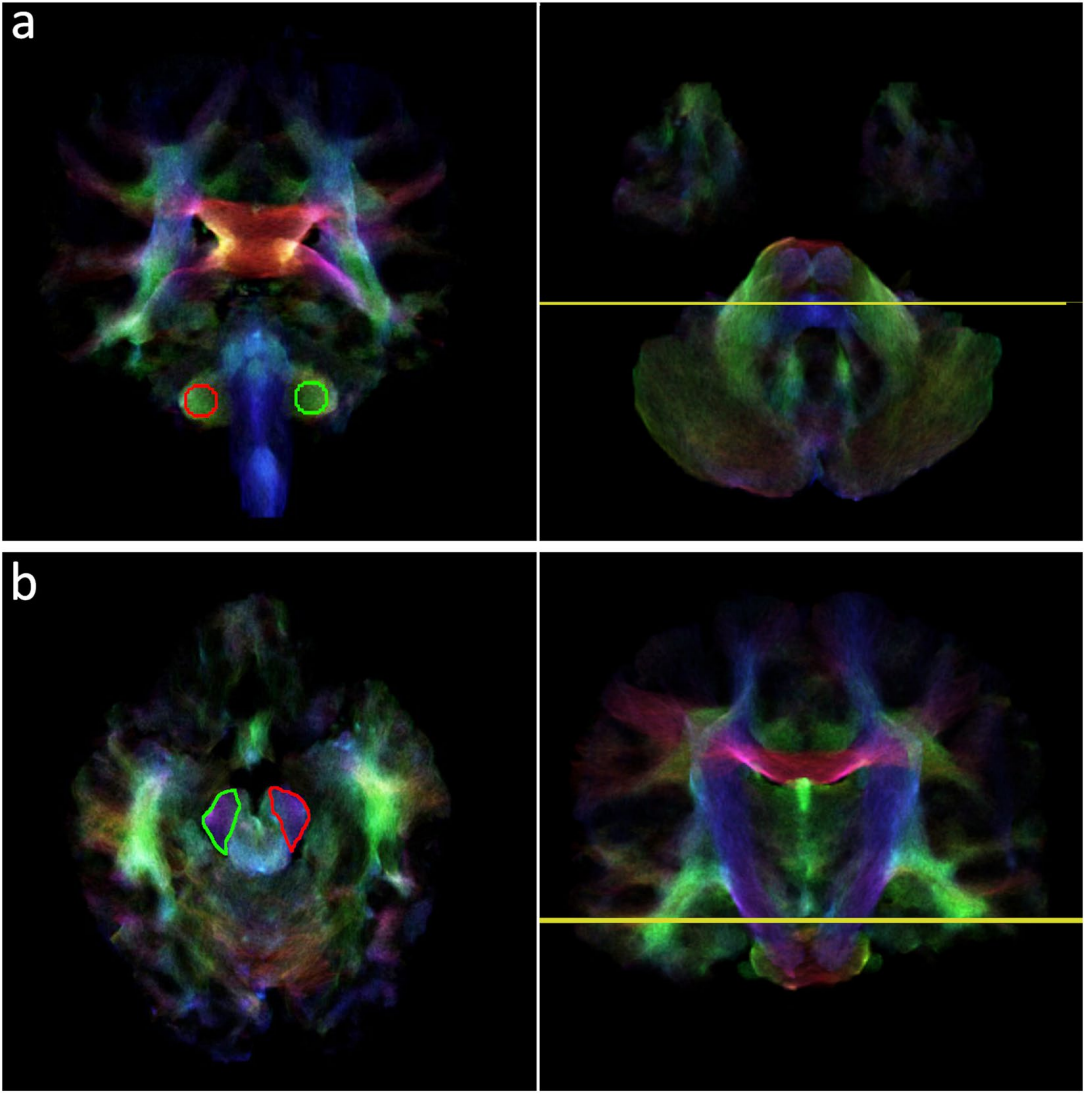
Seed and target ROIs drawn on a colour TDI map. (**a**) Seed ROIs placed in the coronal plane on both right (red) and left (green) middle cerebellar peduncle at the level identified by the yellow line in the axial plane (right panel). (**b**) Target ROIs placed in the axial plane on both right (green) and left (red) cerebral peduncle at the level identified by the yellow line in the coronal plane (right panel). The correspondence between each seed ROI and its contralateral target ROI is represented by the same colour.
- Brain cortices parcellation: The atlas of Brodmann areas (BA)^34^ and of the cerebellum (SUIT)^33^ were aligned to the native space of each subject in order to parcellate cerebral and cerebellar cortices based both on anatomical grounds and on a functional basis.

- Pre processing and data alignment: FSL was used to perform eddy current correction and brain extraction. 3D-T1w volumes were realigned to the diffusion data with FLIRT.
- Whole brain tractography and TDI maps: To obtain TDI maps, whole brain tractography was performed using MRtrix and combining CSD with probabilistic tractography (seed = whole brain, step-size = 0.1 mm, maximum harmonics order = 8, termination criteria: exit the brain or when the CSD fiber-orientation distribution amplitude was < 0.1). Streamlines were randomly seeded throughout the whole brain until 2.5 millions of them were selected^37^. A TDI map was computed assigning to each element of a user-defined 1-mm resolution grid^31^ an intensity proportional to the total number of streamlines passing within it^31^.
- Cortico-ponto-cerebellar pathways: the CPC pathway was reconstructed using the MRtrix algorithm mentioned above. Two hand-drawn ROIs were used as seed and target, as previously described^13,26,27^: the MCP^13^ and the whole contralateral CP for each hemisphere^26,27^. A total of 3,000 streamlines for each subject were selected.
- Seed/target ROIs: High-resolution TDI images were used to define both seed and target ROIs. The seed ROI was defined as a sphere with 2.5 mm radius centered on the MCP in each cerebellar hemisphere and was identified in the coronal plane, as described by Calamante *et al*.^31^ (Fig. 5a). The target ROI was traced on the whole contralateral CP^26^ for each cerebral hemisphere. This structure was identified as the blue area (top-down fiber direction) on the axial plane of colour coded TDI maps (Fig. 5b).

Anatomical parcellation consisted of the following areas:

- Cerebrum: prefrontal cortex, frontal, parietal, temporal, occipital and limbic lobes^34^;
- Cerebellum: anterior lobule, VI lobule, lateral crus I–II, lobules VIIb/VIII and inferior lobule^32^.

**Figure 6:**
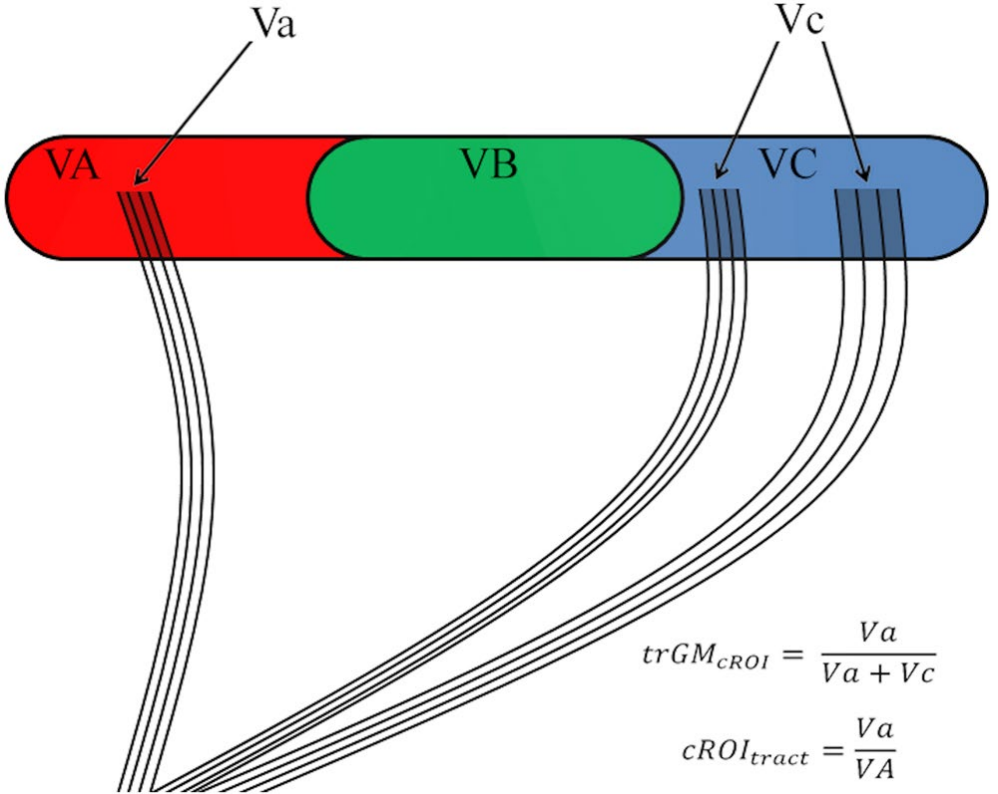
Tractography indices: trGM_cROI_, cROI_tract_. Each colour (red, green, blue) represents a cortical parcellation. VA, VB, VC represent the volume of the red, green and blue parcellation respectively, while Va, Vc represent the volume occupied by the streamlines (black lines in figure) in the red and blue parcellation respectively. The formulas reported refer to the example of the red parcellation.
- Mean cortico-ponto-cerebellar pathway: To assess the consistency of the pathways and for display purposes, the output from all subjects were normalized to MNI space using a non-linear registration algorithm with nearest neighbor interpolation from the FSL library (FNIRT). A mean image was calculated from the binarized pathways (obtained using tracks2prob algorithm from MRtrix) for each subject^65^. Voxels were assigned the count of the number of subjects with that specific voxel included in the mask. The mean image was thresholded to include voxels common to at least 20% of subjects^10^.

Functional parcellation consisted of the following areas:

- Cerebrum: motor, associative, primary sensory, primary auditory and primary visual areas^34^;
- Cerebellum: primary motor, sensorimotor and cognitive/sensory areas^33^.
- Quantification of trGM_cROI_ , cROI_tract_ and TSC: Two metrics were used to quantify the pattern of cerebro-cerebellar connections, summarized in Fig. 6. The trGM_cROI_ index represents the proportion of the tract projecting to a particular ROI, and was computed as the volume of grey matter (GM) belonging to the tract (trGM) in one cortical parcellation, divided by the total trGM across all cortical parcellations. The cROI_tract_ index represents the proportion of a given ROI that is involved in the tract, and was obtained by dividing trGM in one cortical parcellation by the number of voxels within the parcellation itself. The total streamline count (TSC) was used to reflect the number of the streamlines reaching the cortex. TSC was calculated with MRtrix filtering from the tract only the streamlines related to each specific cortical area.
